# Sulforaphane prevention of diabetes-induced aortic damage was associated with the up-regulation of Nrf2 and its down-stream antioxidants

**DOI:** 10.1186/1743-7075-9-84

**Published:** 2012-09-15

**Authors:** Xiao Miao, Yang Bai, Weixia Sun, Wenpeng Cui, Ying Xin, Yuehui Wang, Yi Tan, Lining Miao, Yaowen Fu, Guanfang Su, Lu Cai

**Affiliations:** 1 The Second Hospital of Jilin University, Changchun, China; 2 KCHRI at the Department of Pediatrics, University of Louisville, Louisville, USA; 3 The People’s Hospital of Jilin Province, Changchun, China; 4 The First Hospital of Jilin University, Changchun, China; 5 Normal Bethune Medical College of Jilin University, Changchun, China; 6 Chinese-American Research Institute for Diabetic Complications at Wenzhou Medical College, Wenzhou, China; 7 Departments of Radiation Oncology and Pharmacology and Toxicology, The University of Louisville, Louisville, USA

**Keywords:** Sulforaphane, Nrf2, aorta, Oxidative damage, Vascular inflammation

## Abstract

**Background:**

Oxidative stress plays an important role in diabetes-induced vascular inflammation and pathogenesis. Nuclear factor E2-related factor-2 (Nrf2) is a transcription factor orchestrating antioxidant and cyto-protective responses to oxidative stress. In the present study, we tested whether sulforaphane (SFN) can protect the aorta from diabetes and, if so, whether the aortic protection is associated with up-regulation of Nrf2 and its down-stream antioxidants.

**Methods:**

Type 1 diabetes was induced in FVB mice by multiple low-dose streptozotocin. Diabetic and age-matched control mice were treated with or without SFN at 0.5 mg/kg daily in five days of each week for three months. At the end of 3 months treatment of SFN one set of mice were sacrificed to perform the experimental measurements. The second set of both diabetic and control mice were aged for additional 3 months without further SFN treatment and then sacrificed to perform the experimental measurements. Aortas from these mice were assessed for fibrosis, inflammation, oxidative damage, and Nrf2 expression and transcription by immunohistochemical staining and real-time PCR method, respectively.

**Results:**

Diabetes induced significant increases in oxidative stress and inflammation in the aorta at both 3 and 6 months, and fibrotic response at 6 months. SFN completely prevented these diabetic pathogenic changes and also significantly up-regulated the expression of Nrf2 and its down-stream antioxidants.

**Conclusions:**

These results suggest that diabetes-induced aortic fibrosis, inflammation, and oxidative damage can be prevented by SFN. The aortic protection from diabetes by SFN was associated with the up-regulation of Nrf2 and its downstream antioxidants.

## Background

Systemic complications are the major cause of morbidity and mortality in patients with either Type 1 or Type 2 diabetes. These complications are divided into microvascular and macrovascular disorders [1,2]. The latter includes coronary artery disease, atherosclerosis and peripheral vascular disease. Although glucose control, blood pressure control, lipid lowering, and the blockade of the renin-angiotensin system were used for the treatment of diabetic patients, the development and progression of vascular complications in the patients with diabetes remains unpreventable [1]. Therefore, an effective approach to prevent or delay the development and progression of these lethal complications for diabetic patients are urgently needed.

Increasing evidence indicates that increased production of reactive oxygen or nitrogen species (ROS or RNS) and/or impaired endogenously protective mechanism is the major factor responsible for the development and progression of vascular complications in diabetic patients, although several other mechanisms were also proposed [3-5]. Exogenous supplementation of a single or few antioxidants in clinics often fails to efficiently prevent or treat various complications for diabetic patients; therefore up-regulation of endogenous, multiple antioxidants may be a better approach for the prevention of diabetic cardiovascular complications [6].

The transcriptional factor NFE2-related factor 2 (Nrf2) as one member of the cap’n’collar family is a master regulator of cellular detoxification responses and redox status [7]. Under physiological conditions Nrf2 locates in the cytoplasm and binds to its inhibitor kelch-like ECH-associated protein 1 (KEAP1) [8]. KEAP1 could mediate a rapid ubiquitination and subsequent degradation of Nrf2 by the proteasome [8]. Upon exposure of cells to oxidative stress or electrophilic compounds, Nrf2 is free from KEAP1 and translocates into the nucleus to bind to antioxidant-responsive elements (ARE) in the genes encoding antioxidant enzymes such as NADPH quinoneoxidoreductase (NQO1), heme oxygenase-1 (HO-1), glutathione S-transferase, superoxide dismutase (SOD), catalase, and γ-glutamylcysteine synthetase, increasing their expression to play a role in the detoxification, antioxidant, and anti-inflammatory [7-9]. Nrf2 is appreciated now for its potential prevention of or therapy for diabetic complications [10,11].

Sulforaphane (SFN) is an organosulfur compound that exhibits anticancer and anti-diabetic properties in experimental models, and obtained from cruciferous vegetables such as broccoli, brussels sprouts or cabbages [12]. Therefore, increased consumption of cruciferous vegetables has been associated with a decreased risk of several degenerative and chronic diseases, including cardiovascular disease. SFN has garnered particular interests as an indirect antioxidant due to its extraordinary ability to induce expression of endogenous, multiple enzymes via the up-regulation of Nrf2 function [12].

**Figure 1:**
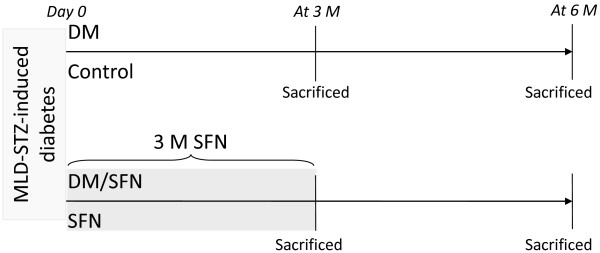
**Outline of experimental procedures.** Day 0 means that hyperglycemia was defined at 7 days after multiple low doses of STZ (MLD-STZ) treatment. SFN: Sulforaphane which was given subcutaneously at 0.5 mg/kg daily in five days of each week for 3 months. Some of these mice were sacrificed immediately at the end of the 3-month SFN treatment. The rest mice were sacrificed at 3 months after the 3-month SFN treatment. DM: diabetes.

Therefore the present study aimed to investigate whether chronic use of SFN can prevent the development of diabetes-induced aortic pathogenesis. To the end, we have used a type 1 diabetic mouse model induced with multiple low-dose streptozotocin (MLD-STZ). Diabetic and age-matched control mice were treated with SFN for 3 months. At the end of 3 months treatment of SFN one set of mice were sacrificed to perform the experimental measurements. The second set of both diabetic and control mice were aged for additional 3 months without further SFN treatment and then sacrificed to perform the experimental measurements, as illustrated in Figure 1.

## Experimental methods

### Animals

FVB male mice, 8–10 weeks of age, were purchased from the Jackson Laboratory (Bar Harbor, Maine) and housed in the University of Louisville Research Resources Center at 22°C with a 12 h light/dark cycle with free access to standard rodent chow and tap water. All experimental procedures for these animals were approved by the Institutional Animal Care and Use Committee of the University of Louisville, which is compliant with the Guide for the Care and Use of Laboratory Animals published by the US National Institutes of Health (NIH Publication No. 85–23, revised 1996).

For type 1 diabetic mouse model, mice were injected intraperitoneally with MLD-STZ (Sigma-Aldich, St. Louis, MO), dissolved in 0.1 M sodium citrate buffer (pH4.5), at 50 mg/kg body-weight daily for 5 days while age-matched control mice were received multiple injections of the same sodium citrate buffer. Five days after the last injection, mice with hyperglycemia (blood glucose levels ≥ 250 mg/dl) were considered as diabetic as before [13]. SFN (Sigma-Aldich) was given subcutaneously at 0.5 mg/kg daily in five days of each week for 3 months. At the end of 3-month SFN treatment one set of mice were sacrificed to perform the experimental measurements. The second set of both diabetic and control mice were aged for additional 3 months without further SFN treatment and then sacrificed to perform the experimental measurements, as illustrated in Figure 1. Dose of SFN was selected based on published information [14]. Mice were randomly allocated into four groups (n = 6 at least per group): Control, SFN, diabetes (DM) and DM plus SFN (DM/SFN), as outlined in Figure 1. Since SFN was dissolved in dimethyl sulfoxide (DMSO) and diluted in PBS, mice serving as vehicle controls were given the same volume of PBS (1% DMSO).

### Aorta preparation and histopathological examination

After anesthesia, thorax was opened and descending thoracic aortas were isolated carefully and cleaned of surrounding fat and connective tissue. Aortas tissues were fixed in 10% buffered formalin and then cut into ring segments (2 – 3 mm in length) for being dehydrated in graded alcohol series, cleared with xylene, embedded in paraffin, and sectioned at 5 μm thickness for pathological and immunohistochemical staining.

Paraffin sections (5 μm thickness) from aortic tissues were dewaxed and incubated with 1X Target Retrieval Solution (Dako, Carpinteria, CA) in a microwave oven for 15 min at 98°C for antigen retrieval, with 3% hydrogen peroxide for 15 min at room temperature, and then with 5% animal serum for 30 min, respectively. These sections were incubated with primary antibodies against connective tissue growth factor (CTGF) and transforming growth factor (TGF)-β1 at 1:100 dilution (Santa Cruz Biotechnology, Santa Cruz, CA), 3-nitrotyrosine (3-NT) at 1:400 dilution (Millipore, Billerica, CA), 4-hydroxy-2-nonenal (4-HNE) at 1:400 dilution (Alpha Diagnostic International, San Antonio, TX), plasminogen activator inhibitor-1 (PAI-1) at 1:100 dilution (BD Bioscience, San Jose, CA), TNF-α (Abcam, Cambridge, MA)at 1:100 dilution, Nrf2at 1:100 dilution, and Cu-Zn superoxide dismutase-1 (SOD-1) at 1:400 dilution (both from Santa Cruz Biotechnology) overnight at 4°C. After sections were washed with PBS, they were incubated with horseradish peroxidase conjugated secondary antibodies (1:300 – 400 dilutions with PBS) for 2 h in room temperature. For the development of color, sections were treated with peroxidase substrate DAB kit (Vector Laboratories, Inc. Burlingame, CA) and counterstained with hematoxylin.

### Sirius-red staining for collagen

Aortic fibrosis was reflected by Sirius-red staining for collagen, as described in our previous study [15]. Briefly, 5 μm tissue sections were used for Sirius-red staining with 0.1% Sirius-red F3BA and 0.25% Fast Green FCF. Sections stained for Sirius-red then were assessed for the proportion of collagen using a Nikon Eclipse E600 microscopy system

### Real-time qPCR

Collected aortas were snap frozen in liquid nitrogen and kept at - 80°C. Total RNA was extracted using the TRIzol Reagent (Invitrogen, USA). RNA concentrations and purities were quantified using a Nanodrop ND-1000 spectrophotometer. First-strand complimentary DNA (cDNA) was synthesized from total RNA according to manufacturer’s protocol from the RNA PCR kit (Promega, Madison, WI). Reverse transcription was performed using 0.5 μg of total RNA in 12.5 μl of the solution containing 4 μl 25 mM MgCl_2_, 4 μl AMV reverse transcriptase 5 X buffer, 2 μl dNTP, 0.5 μl RNase inhibitor, 1 μl of AMV reverse transcriptase, and 1 μl of oligo dT primer, which were added with nuclease-free water to make a final volume of 20 μl. Reaction system was run at 42°C for 50 min and 95°C for 5 min. Primers of NQO1, HO-1, SOD-1, and β-actin were purchased from Applied Biosystems (Carlsbad, CA). Real-time quantitative PCR (qPCR) was carried out in a 20 μl reaction buffer that included 10 μl of TaqMan Universal PCR Master Mix, 1 μl of primer, 9 μl of cDNA with the ABI 7300 Real-Time PCR system. The fluorescence intensity of each sample was measured at each temperature change to monitor amplification of the target gene. The comparative cycle time (CT) was used to determine fold differences between samples.

### Statistical analysis

Data were collected from several animals and presented as means ± SD (n = 6). We used Image Pro Plus 6.0 software and a IOD (integrated optical density) divided area method to identify the positive staining area of interest. Comparisons were performed by one-way ANOVA for the different groups, followed by post hoc pairwise repetitive comparisons using Tukey’s test with Origin 7.5 Lab data analysis and graphing software. Statistical significance was considered at *P* <0.05.

## Results

### Preventive effect of SFN on diabetes-induced aortic fibrosis

**Figure 2:**
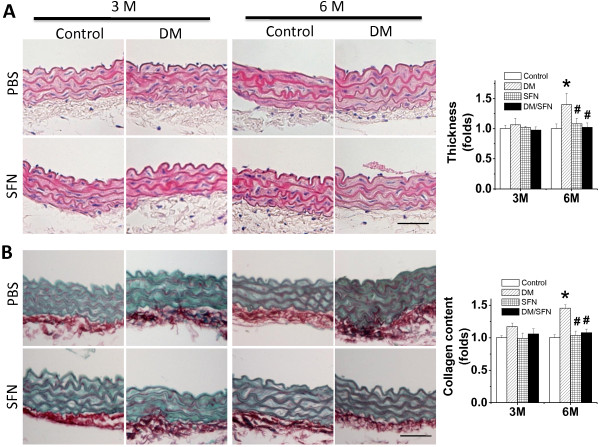
**Preventive effect of SFN on diabetes-induced aortic pathological changes.** The pathogenic changes of aortas were examined by H&E staining (**A**), and Sirius-red staining for collagen accumulation (**B**), followed with semi-quantitative analysis. Data were presented as means ± SD (n = 6). *, p < 0.05 vs. control; #, p < 0.05 vs. DM. Bar = 50 μM.

MLD-STZ-induced diabetic and age-matched control mice were treated with SFN for 3 months. At the end of 3 months treatment of SFN one set of mice were sacrificed to perform the experimental measurements. The second set of both diabetic and control mice were kept for additional 3 months without further SFN treatment and then sacrificed to perform the experimental measurements, as illustrated in Figure 1. Aortas were examined pathologically with H&E staining (Figure 2A), which showed the increase in tunic media thickness slightly at 3 months and significantly at 6 months of diabetes. Similarly Sirius-red staining revealed an increased collagen accumulation in aortic tunica media of diabetic mice at 6 months (Figure 2B). Both pathological alterations were completely prevented by SFN treatment, which was observed not only at the end of the 3-month SFN treatment, but also 3 months after the 3-month SFN treatment.

**Figure 3:**
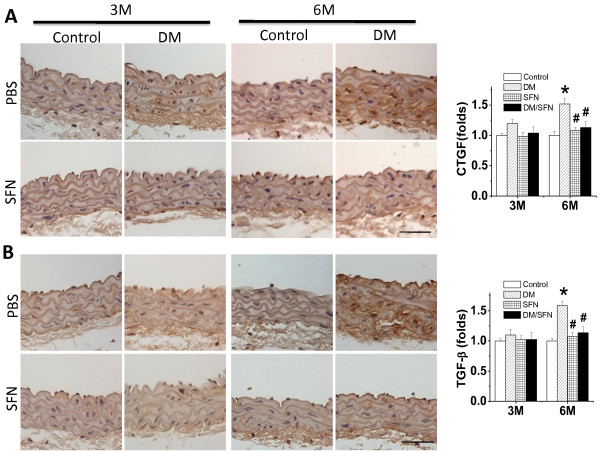
**Preventive effect of SFN on diabetes-induced aortic fibrosis.** Aortic fibrosis was examined by immunohistochemical staining for the expression of CTGF (**A**) and TGF-β1 (**B**), followed with semi-quantitative analysis. Data were presented as means ± SD (n = 6). *, p < 0.05 vs. control; #, p < 0.05 vs. DM. Bar = 50 μM.

To further examine the preventive effect of SFN on diabetes-induced aortic fibrosis, immunohistochemical staining showed the increased expression of two important pro-fibrotic mediators, CTGF and TGF-β1 (Figure 3A,B), in aortic tunica media of DM mice, particularly at the 6 month time-point. Treatment with SFN can completely prevent the fibrotic response in the aortas induced by diabetes (i.e.: DM/SFN group).

### Preventive effect of SFN on diabetes-induced aortic inflammation and oxidative damage

**Figure 4:**
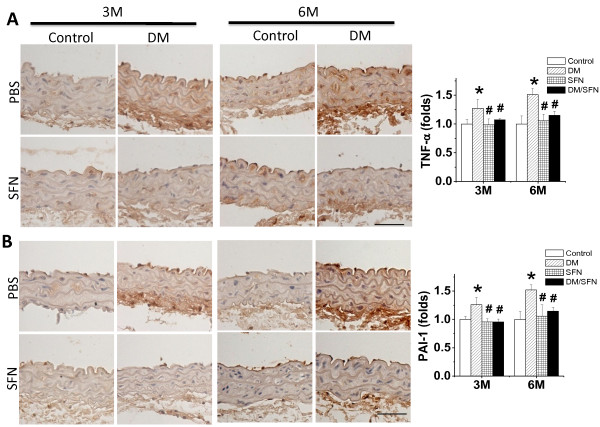
**Preventive effect of SFN on diabetes-induced aortic inflammation.** Aortic inflammation was examined by immunohistochemical staining for the expressions of TNF-α (**A**) and PAI-1 (**B**), followed by semi-quantitative analysis. Data were presented as means ± SD (n = 6).*, p < 0.05 vs. control; #, p < 0.05 vs. DM. Bar = 50 μM.

Since both inflammation and oxidative damage are major causative factors for the fibrotic response, the expression of TNF-α (Figure 4A) and PAI-1 (Figure 4B) as indices of inflammation was examined with immunohistochemical staining. Diabetes was found to induce a progressive increase in aortic inflammation, an effect that was completely prevented by 3-month treatment with SFN.

**Figure 5:**
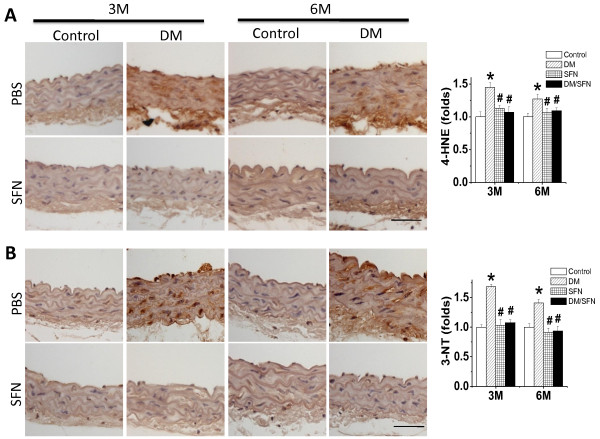
**Preventive effect of SFN on diabetes-induced aortic oxidative damage.** The oxidative damage was examined by immunohistochemical staining for the accumulation of 4-HNE (**A**) and 3-NT (**B**), followed with semi-quantitative analysis. Data were presented as means ± SD (n = 6).*, p < 0.05 vs. control; #, p < 0.05 vs. DM. Bar = 50 μM.

Oxidative damage was detected by examining the accumulation of 4-HNE and 3-NT as indices of lipid peroxidation and protein nitration, respectively. Figure 5 shows that diabetes significantly increased aortic accumulation of 4-HNE and 3-NT, both which were found to be more significant at 3 months than that at 6 months. However, treatment with SFN for 3 months completely prevented the oxidative damage at both time-points.

### SFN up-regulated Nrf2 expression and transcription

**Figure 6:**
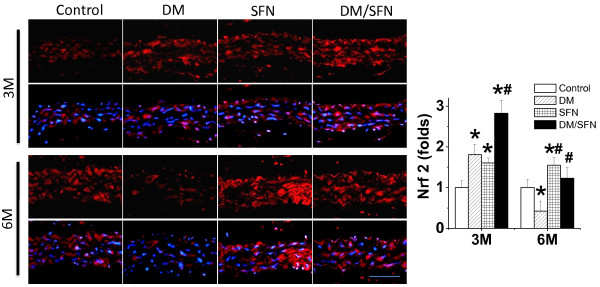
**Effects of SFN on aortic expression of Nrf2.** Aortic expression of Nrf2 was examined by immunohistochemical staining for the expression of Nrf2 in aortic tunica media with semi-quantitative analysis (Staining for 3 month & 6 month sections were not at same time so that control staining intensity was different between two time-points). Data were presented as means ± SD (n = 6).*, p < 0.05 vs. control; #, p < 0.05 vs. DM. Bar = 50 μM.

Above results showed that SFN can protect diabetic induction of aortic oxidative stress and damage, inflammation, and remodeling. Since SFN is an Nrf2 activator, whether the SFN prevention of diabetes-induced these pathogenic changes are associated with up-regulation of Nrf2 was examined first by measuring Nrf2 expression with immunofluorescent staining (Figure 6). It was shown that Nrf2expression significantly increased in the aorta of SFN-treated control mice both at 3 months (i.e.: at the end of 3-month SFN treatment) and 6 months (3 months after the end of 3-month SFN treatment) of diabetes. Diabetes also significantly increased aortic Nrf2 expression at 3 months, but significantly decreased aortic Nrf2 expression at 6 months of diabetes. There was significantly synergistic increase in the aortic Nrf2 expression in DM/SFN group at 3-month time-point compared to DM group or SFN group.

**Figure 7:**
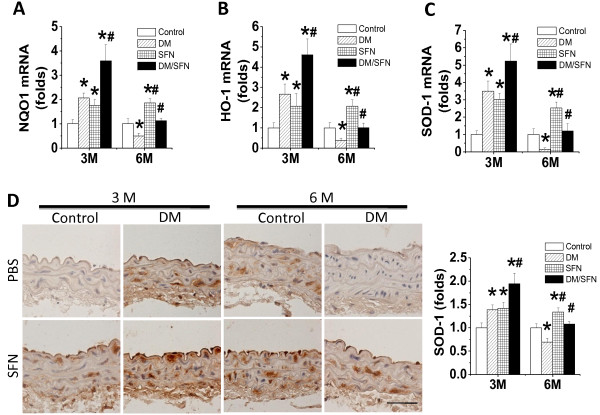
**Effects of SFN on aortic expression of Nrf2 downstream genes.** Aortic expression of Nrf2 down-stream genes NQO1 (**A**), HO-1 (**B**), and SOD-1 (**C**) at mRNA levels was measured by real-time PCR. Immunohistochemical staining was used to measure the expression of SOD-1 at protein levels in aortic tunica media (**D**) with semi-quantitative analysis (Staining for 3 month & 6 month sections were not performed at same time so that control staining intensity was different between two time-points). Data were presented as means ± SD (n = 6).*, p < 0.05 vs. control; #, p < 0.05 vs. DM. Bar = 50 μM.

Immunofluorescent staining also showed that SFN can increase the nuclear accumulation of Nrf2 in the control or diabetic aortas (Figure 6), suggesting the activation of Nrf2 transcriptional function. Therefore we further explored Nrf2 function by examining the expression of its downstream anti-oxidative genes, NQO1 (Figure 7A), HO-1 (Figure 7B), and SOD-1 (Figure 7C). Expression of these genes at mRNA level was significantly increased in the aorta of SFN-treated control mice at both 3 months and 6 months; Diabetes significantly increased at 3 months, but significantly decreased at 6 months, aortic mRNA expression of these genes. However, aortic mRNA expression of these genes was significantly higher in DM/SFN mice than DM mice at both 3 months and 6 months of diabetes.

Immunohistochemical staining further confirmed: (1) SFN-treatment significantly increased the aortic SOD-1 expression in the control groups at both 3 months and 6 months; (2) Diabetes significantly increased at 3 months, and significantly decreased at 6 months, the aortic expression of SOD-1 protein; (3) Aortic expression of SOD-1 protein was significantly higher in DM/SFN mice than DM mice at both 3 months and 6 months of diabetes (Figure 7D).

## Discussion

The present study provided the first experimental evidence to show the significant protection of the aorta by SFN against diabetes-induced damage in MLD-STZ-induced type 1 diabetic mouse model, which is associated with the up-regulation of aortic Nrf2 expression and transcription. Most importantly, we demonstrated for the first time that SFN afford aortic protection can be sustained for long-time (3 months) after stopping the treatment.

Using MLD-STZ-induced type 1 diabetic mouse model here the diabetic damage to the aorta was successfully developed, which is reflected by significantly progressive increases of aortic fibrosis or remodeling from a slight increase (p > 0.05) at 3 months of diabetes to a significant increase (P < 0.05) at 6 months of diabetes (Figures 2,3). We also demonstrated that aortic expression of Nrf2 and its downstream genes in DM group was significantly increased at 3 months, but significantly decreased at 6 months of diabetes (Figures. 6,7).

It is known that Nrf2 expression and transcription in cultured cells in vitro and tissues *in vivo* are increased in response to oxidative stress [16-18]. There is strong evidence showing that aging is associated with vascular oxidative stress, which has been causally linked to the development of cardiovascular diseases. Ungvari *et al.*[19] found that in the aorta of Fischer 344 × Brown Norway rats, aging results in a progressive increase in superoxide and a decrease in Nrf2 mRNA and protein expression that was associated with decreases in the nuclear Nrf2 activity and the expression of Nrf2 down-stream genes NOQ1 and HO-1. They found an inverse relationship between vascular expression of Nrf2 target genes and age-related increases in the expression of the NF-κB target genes ICAM-1 and IL-6. This study suggested that aging is associated with Nrf2 dysfunction in the vasculature, which likely exacerbates age-related cellular oxidative stress and increases sensitivity of aged vessels to oxidative stress-induced cellular damage. In another study, Ungvari *et al.* also found that high-fat diet-induced increases in endothelia ROS levels and endothelial dysfunction were significantly greater in Nrf2-KO mice than wild-type mice [20]. These results suggest that adaptive activation of the Nrf2 pathway confers endothelial protection under obese and/or diabetic conditions. In agreement with these findings, we also found here that Nrf2 expression in the aorta was significantly up-regulated in the diabetic mice at 3 months along with no significant damage, but significantly down-regulated in the diabetic mice at 6 months along with significant aortic damage.

The most innovative finding of the present study is that treatment of diabetic mice for the first 3 months provided an aortic protection from diabetes, which could be observed not only at the end of the treatment, but also at 3 months after stopping SFN treatment. In terms of the mechanism by which SFN-induced aortic protection can be sustained for long time at least for 3 months it remains unclear. However, whether the up-regulation of Nrf2 expression and transcription by SFN is mediated by epigenetic modification may be a potential mechanism. The epigenome is comprised of all chromatin modifications including post translational histone modification, expression control via miRNAs and the methylation of cytosine within DNA. Modifications of these epigenetic marks not only allow cells and organisms to quickly respond to changing environmental stimuli but also confer the ability of the cell to "memorize" these encounters. Methylation of CpG repeats in the upstream/promoter regions of genes is an established mechanism of gene silencing in many cell types. DNA methylation results in the recruitment of histone deacetylases (HDACs) to promoter regions, thereby repressing expression of genes. For instance in transgenic adenocarcinoma of the mouse prostate (TRAMP) model, the suppressed expression of Nrf2 gene in TRAMP tumors was because the specific CpG sites of Nrf2 gene promoter region were hypermethylated [21-23]. Therefore, anything that can inhibit DNA methyltransferases activity has potential to demethylate the CpG sites of Nrf2 gene promoter region, leading to repression of Nrf2 gene [22,23]. SFN was recently reported to have the function to demethylate certain hypermethylated sites to repress the target gene expression [24]. Therefore, SFN may repress Nrf2 expression under diabetic condition, which may become the sustained (memorized) up-regulation of Nrf2 gene and aortic protection from diabetes.

## Conclusions

We have investigated whether SFN as one of Nrf2 activators can protect the aorta from diabetes using a Type 1 diabetes model. We treated diabetic and age-matched control mice with SFN at 0.5 mg/kg for 3 months, resulting in a significant prevention of diabetes-induced progression of aortic pathogenic damage. The aortic protection by SFN treatment at early stage was still remarkable, examined at the late stage of the diabetes, i.e.: at 3 months after stopping SFN treatment. The aortic protection from diabetes was accompanied with a significant up-regulation of Nrf2 expression and function. These results suggest that diabetic damage to the aorta can be prevented by SFN most likely via up-regulation of Nrf2 expression and function.

## Competing interests

The authors declare that they have no conflict of interest.

## Authors’ contributions

XM, YB, WC, WS, YW, YX and YT researched data. XM, YT, YF, LM, and GS reviewed the article. YT, GS and LC contributed initial discussion of and overseeing the project. L.C. wrote, edited, and reviewed the article. All authors read and approved the final manuscript.
